# The Case of Dr. Lamont

**Published:** 1899-07-22

**Authors:** 


### The Case of Dr. Lamont.

The parochial Jack in Office, even as seen in England, occasionally furnishes examples of vindictiveness and stupidity, but these fine qualities, if we may judge from the disclosures made in the course of a debate in the House of Commons on Friday, are to be found in their most complete development in the western islands of Scotland. It appears that at some recent period, the date of which was not precisely given, an epidemic said to be of typhus fever was raging in South Uist, and that, when this epidemic was at its height, Dr. Lamont was appointed medical officer to the island, and at once entered upon his duties with no common energy, assiduity, and success. In the words of Sir C. Cameron, he "showed such indefatigable zeal and such disregard for personal danger, travelling day and night over his wide parish, that he received a special letter from the Local Government Board conveying to him the Board's high appreciation of his conduct." In the words of Dr. Farquharson, "Dr. Lamont had attended the victims of typhus fever when others fled, he had watched the dying moments of the sufferers, had put them in their coffins, and he was not sure that he had not actually buried them." Certain officials, whose own cowardice had very properly been denounced by those in authority, then "entered into a most mean and wretched conspiracy to hound this brave man out of office." He had insisted upon closing certain Board schools during a prevalence of scarlatina, schools which were opened again by the Board; and, between the School Board and the Parish Council, it was determined that the too upright and too capable doctor should be dismissed. According to Dr. Farquharson, "a minister of religion" was enlisted in the sacred causes of filth and of disease, "and actually denounced Dr. Lamont from the open grave of one of his patients in such vigorous terms that no person dared to consult him, so that a large number of people actually died because under the sway of this priestly despotism they had gone without medical assistance." There appears to be no central authority in Scotland able to extend to medical officers the protection which they receive from the Local Government Board in England, and the Parish Council was therefore able to effect Dr. Lamont's dismissal. Not content with this, his enemies are said to have trumped up charges that he had given false vaccination certificates, and a prosecution was commenced against him, under which he was arrested in the middle of a Saturday night when in bed in his mother's house in Glasgow, detained in the police cells until Monday, and then taken in custody to Lochmaddy, a journey of one day and two nights, in order to meet the accusation. The only foundation for it was that it had been a custom, doubtless a bad one, in the locality, to give certificates of successful vaccination on a description of the state of the arm furnished by a parent or other responsible person, and without an examination which would often have involved a very long journey either for the doctor or for the infant. The custom was indefensible, but it had prevailed, and to make it the foundation of criminal proceedings in the form in which they were taken was absurd. Dr. Lamont was instantly acquitted by the jury before whom he was tried; and his case was brought before the House of Commons with a view to obtaining for him some compensation for the manner in which he had been treated, and also in order to obtain the punishment of an official called the "Procurator Fiscal," who had caused his arrest and imprisonment in circumstances which afforded no reasonable justification for such a course. A Procurator Fiscal, in Scotland, is a local officer charged with the prosecution of crimes, and, like a coroner in England, with inquiry into the causes of death. He is appointed by the Sheriff; and the Lord Advocate told the House that, although he could and would censure his conduct in the case, he had no power to remove him, or, presumably, to punish him in any way. If his course of action were correctly described in the debate, he is probably a person who would take but small heed of censure; and it is difficult to avoid some feeling of compassion for the luckless Hebrideans among whom his lot is cast. The only satisfactory part of the sordid story is that both sides of the House were unanimous in their praise of Dr. Lamont, and in their denunciation of the unmerited persecution to which he had been subjected; and that, after a sympathetic speech from Mr. Balfour, the Lord Advocate announced that the question of giving compensation would be seriously considered by the Government. Still, the matter ought not to end here. It is true, as Mr. Balfour said, that the feeling of the House was strongly in favour of Dr. Lamont, and that the action taken against him was viewed with distrust, if not with something much stronger. It is, unfortunately, also true that no vote of the House could do anything "either to protect the medical profession generally or Dr. Lamont in particular from any evil consequences arising from the due, courageous, and disinterested exercise of duty in circumstances of exceptional difficulty." This is the point to which the attention alike of the Government and of Scotch members should be directed, and it seems to be one which calls imperatively for legislation.

